# Incidence, Survival Analysis and Future Perspective of Primary Peritoneal Mesothelioma (PPM): A Population-Based Study from SEER Database

**DOI:** 10.3390/cancers14040942

**Published:** 2022-02-14

**Authors:** Asad Ullah, Abdul Waheed, Jaffar Khan, Ankita Mishra, Bisma Tareen, Noor Nama, Nabin Raj Karki, Muhammad Saleem Panezai, Luis Velasquez Zarate, Joseph White, Frederick D. Cason, Nathaniel Matolo, Subhasis Misra, Nagla Abdel Karim

**Affiliations:** 1 Georgia Cancer Center, Medical College of Georgia, Augusta University, Augusta, GA 30912, USA; aullah@augusta.edu (A.U.); nkarki@augusta.edu (N.R.K.); luchovlz352@gmail.com (L.V.Z.); jwhite3@augusta.edu (J.W.); 2 Department of Surgery, San Joaquin General Hospital, French Camp, CA 95231, USA; awaheed@sjgh.org (A.W.); fcason@sjgh.org (F.D.C.); nmatolo@sjgh.org (N.M.); 3 Department of Pathology, Indiana University School of Medicine, Indianapolis, IN 46202, USA; khanja@iu.edu; 4 Department of Surgery, Brandon Regional Hospital, Brandon, FL 33511, USA; ankita.mishra@hcahealthcare.com (A.M.); subhasis.misra@hcahealthcare.com (S.M.); 5 Department of Medicine, Bolan Medical College, Quetta 87300, Pakistan; bismatareen@hotmail.com (B.T.); noortaqwa2016@gmail.com (N.N.); saleem_panezai@yahoo.com (M.S.P.)

**Keywords:** SEER, mesothelioma, peritoneal, HIPEC, radiation, surgery

## Abstract

**Simple Summary:**

Mesotheliomas arising from the lining of the mesothelial cells of the peritoneum are rare. However, they are strongly linked to asbestos exposure, similar to the relatively well-known pleural mesotheliomas. It is slightly more common in men than in women, with the majority of cases seen in Caucasians older than 50 years. Distant spread of tumor, size > 4 cm, and negative lymph node status were observed in our study among the patients with evaluable data. Optimal outcomes are achieved when patients are treated in specialized centers with surgical debulking followed by heated chemotherapy administered intraoperatively. Systemic chemotherapy and radiation are options for the selected patient groups. Patients with poorly differentiated large tumors (>4 cm), Caucasian race, and distant spread of disease outside the abdominal cavity have worse prognosis.

**Abstract:**

Background: Primary peritoneal mesothelioma (PPM) is a rare and aggressive tumor arising from the visceral and parietal peritoneum. The diagnosis and treatment of PPM are often delayed because of non-specific clinical presentation, and the prognosis is worse. The current study investigated the demographic, clinical, and pathological factors affecting patient prognosis and survival in PPM. Methods: Demographic and clinical data of 1998 patients with PPM were extracted from the Surveillance Epidemiology and End Results (SEER) database (1975–2016). The chi-square test, paired *t*-test, and multivariate analysis were used to analyze the data. Results: The majority of PPM patients were male (56.2%, *p* < 0.005) and Caucasian (90.4%, *p* < 0.005, with a mean age of diagnosis was 69 ± 13 years. The grading, histological, and tumor size information were classified as “Unknown” in most of the cases, but when available, poorly differentiated tumors (8.7%), malignant mesothelioma, not otherwise specified (63.4%) and tumors > 4 cm in size (8%), respectively, were most common, *p* < 0.005. Chemotherapy was administered to 50.6% of patients, followed by resection (29.2%) and radiation (1.5%), *p* < 0.001. The cohort of PPM had a five-year overall survival of 20.3% (±1.1), compared to 43.5% (±5.9), 25.9% (± 8.4), and 18.7% (±1.6) for those with surgery, radiation, or chemotherapy alone, respectively. Poor differentiation (OR = 4.2, CI = 3.3–4.9), tumor size > 4 cm (OR = 3.9, CI = 3.2–4.5), Caucasian race (OR = 2.9, CI = 2.6–4.4), and distant SEER stage (OR = 2.5, CI = 1.1–3.2) were all linked with increased mortality (*p* < 0.001). Conclusion: An extremely rare and aggressive peritoneal tumor, PPM may be difficult to identify at the time of diagnosis. Radiation therapy likely to have a limited function in the treatment of this condition, with surgery and chemotherapy being the primary choices. All PPM patients should be enrolled in a nationwide registry to improve our understanding of the pathogenesis and identify factors affecting survival.

## 1. Introduction

Malignant mesothelioma (MM) is an aggressive and lethal disease. It affects pleural and peritoneal membranes and is associated with asbestos exposure [1,2]. PPM comprises 30% of all mesotheliomas, second to pleural mesothelioma [3,4]. Approximately 85% of all mesotheliomas are associated with asbestos exposure in males [5].

Due to nonspecific clinical features and an indolent course, diagnosis is often delayed; if an adequate tumor specimen is not available, such as that obtained by ascitic fluid sampling, it may be mistaken for other benign or malignant abdominal processes [6].

Suspected cases were mostly advanced. PPM presents with ascites, significant weight loss, fatigue, anorexia, palpable abdominal mass, and signs of intestinal obstruction [7]. Likewise, PPM has different variants, and the precise diagnosis of PPM with the subtype can only be made with tissue biopsy using special stains [8]. Although no specific imaging has been beneficial in diagnosing PPM, abdominal imaging, particularly multidetector computed tomography (MDCT), helps in further evaluation and extent of the tumor [9,10].

Additionally, no specific guidelines exist for PPM management; however, cytoreductive surgery and hyperthermic intraperitoneal chemotherapy (HIPEC) are recommended in most eligible patients [11]. The role of systemic chemotherapy is limited to patients who are not good candidates for cytoreductive surgery and HIPEC [11]. Even with optimum treatment, the overall survival of PPM is dismal [11]. So far, most data regarding PPM are based on a few case reports and case series. To better understand this rare entity, we conducted an updated population-based outcome study using data from the SEER database.

## 2. Materials and Methods

The Surveillance, Epidemiology, and End Result (SEER) database, SEER Stat program version 8.0.4 (https://seer.cancer.gov/seerstat/, accessed on 12 January 2022), was used to retrieve the data for the current study (1975–2016), and individuals with histologically proven PPM were recognized and imported to IBM SPSS^®^v20.2 (IBM Corp, Armonk, NY, USA) for further analysis. Both anatomical codes (C48.1, C48.2) and histological codes (9050/3, 9051/3, 9052/3, 9053/3, and 9055/3) were used to abstract the data. Significant variables included age, gender, race, tumor stage, and type of treatment received.

Moreover, patients without histological confirmation and those diagnosed with in situ cancer were excluded. The current study looked at overall survival, mortality, and 1-, 2-, and 5-year cancer survival. For categorical variables, the chi-square test was utilized, while for continuous variables, the paired t-test and ANOVA were used. Statistical significance was defined at *p* < 0.05 for multivariable analysis.

## 3. Results

Data from 1998 patients were extracted. Patient’s tumor and survival characteristics were observed in the entire cohort.

### 3.1. Demographical Characteristics of Entire Cohort

With an overall mean age at diagnosis of 69 ± 13 years, PPM patients were more likely to be male (*n* = 1122; 56.2%), followed by women (*n* = 876; 43.8%), with a male: female (M: F) ratio of 1:3; *p* < 0.005. Caucasians were the predominant race affected by PPM (*n* = 1806; 90.4%), followed by African Americans (*n* = 101; 5%), and others (*n* = 85; 4.3%); *p* < 0.005. Racial data were not available (*n* = 6; 0.3%); Table 1.

### 3.2. Tumor Characteristics (Histological and Grading)

Grading information and histological information were not available for most patients (*n* = 1649; 82.5%). When available, poorly differentiated tumors (Grade 3) (*n* = 173; 49.6%) were the most common grade, followed by well-differentiated (Grade 1) (*n* = 106; 5.3%), and moderately differentiated (Grade 2) (*n* = 37; 1.9%), *p* < 0.005.

Similarly, the histological type was not specified in most of the cases (*n* = 1266; 63.4%), but when the information was available, most PPMs were of epithelioid type (*n* = 616; 30.8%), followed by biphasic PPM (*n* = 67; 3.3%), fibrous PPM (*n* = 48; 2.4%), and multicystic PPM (*n* = 1; 0.05%), *p* < 0.005; Table 2.

### 3.3. Tumor Size and Extent Characteristics

Data regarding tumor size were not available for most of the patients (*n* = 1785; 89.3%). When available, most of PPMs were >4 cm in size (*n* = 160; 8%), followed by 2–4 cm in size (*n* = 32; 1.60%), and <2 cm in size (*n* = 21, 1.05%), *p* < 0.005 (Table 3)

Similarly, most of the data regarding the extent of the disease were classified as unknown (*n* = 889; 44.50%), whereas when specific information was provided, most of the PPM presented with distant spread (*n* = 591; 29.60%), followed by those with regional spread (*n* = 182, 9.10%), and localized extent (*n* = 166; 8.30%), *p* < 0.001. Most patients with PPM had lymph node-negative status (*n* = 1431; 71.60%), followed an unknown status (*n* = 445; 22.30%), and lymph node-positive status (*n* = 122, 6.10%), *p* < 0.001 (Table 3).

### 3.4. Treatment Characteristics

In those patients who received any type of treatment, the most frequent regimen used was chemotherapy (*n* = 1010; 50.6%), followed by surgical resection (*n* = 584; 29.2%), and radiation therapy (*n* = 29; 1.5%), and the treatment data of (*n* = 375; 18.7%) patients were missing; *p* < 0.001. The chemotherapy group received both HIPEC and systemic chemotherapy regimens (Table 4).

### 3.5. Survival Characteristics

For the entire cohort, the five-year survival rate was found to be 20.3% (±1.1), whereas those who underwent surgery alone, radiotherapy alone, and chemotherapy alone had five-year survival rates of 43% (±5.9), 25.9% (±8.4), and 18.7% (±1.6), *p* < 0.037) (Table 5).

### 3.6. Multivariable Analysis

Multivariable analysis revealed that poor differentiation (OR = 4.2, CI = 3.3–4.9), tumor size > 4 cm (OR = 3.9, CI = 3.2–4.5), Caucasian race (OR = 2.9, CI = 2.6–4.4), and distant SEER stage (OR = 2.5, CI = 1.1–3.2) were all linked with increased mortality (*p* < 0.001) (Table 6).

## 4. Discussion

The World Health Organization (WHO) classifies mesothelioma into three histologic subtypes; epithelioid, sarcomatoid, and biphasic. The epithelioid type is further divided into papillary, tubulopapillary, acinar, adenomatoid, and solid types. The epithelioid type resembles normal mesothelial cells with papillary and tubolopapillary architectures with minimal cellular atypia. The sarcomatoid subtype comprises of spindle cells with malignant osteoid and, chondroid elements. The biphasic subtype of mesothelioma contains both epithelioid and sarcomatoid components [10]. Along with tumor node metastasis (TNM), the staging peritoneal cancer index (PCI) is a measure of disease spread. PCI was scored in 13 abdominal regions for tumor size and distribution. PCI for peritoneal spread of the disease is evaluated using either laparotomy or computed tomography. A high PCI score is associated with a worse prognosis [12]. However, the subclassification of epithelioid mesothelioma and data on the peritoneal cancer index are not available in the SEER registry.

Peritoneal mesotheliomas are fatal neoplasms, with a median survival of 6–12 months [13]. Due to variability and vague presentation, a deeper characterization of the disease is indispensable to advance our understanding. In addition to describing epidemiological characteristics, we investigated contributing factors, such as pathological and clinical factors, that impact the prognosis and survival of patients with PPM through this population-based study from 1975 to 2016 using the SEER database.

PPM primarily affects male patients in their seventh decade of life. To better analyze the age-adjusted incidence rate of PPM in both men and women, Moolgavkar et al. conducted a SEER database study from 1973–2005 [14]. They reported an age-adjusted PPM rate in men to be 1.2 per million person-years and 0.8 per million person-years in women [14]. Consistent with these findings, we report that 56.2% of patients diagnosed with PPM were men, with an average age of 69 years at the time of diagnosis. Moreover, similar to our extensive database study, age at diagnosis has been shown to be a predictor of survival in previous studies, with patients older than 65 years having poor median overall survival [15].

Although a few non-asbestos-related mesothelioma cases have been reported, prolonged asbestos exposure has been linked to PPM development [8,16,17]. Asbestos toxicity generates reactive oxygen species via oxidative stress, causing genomic instability and DNA damage [18]. The molecular changes in PPM have not been well established; however, in 40–70% of PPM patients, loss of 9p, including cyclin-dependent kinase inhibitor 2A (CDKN2A), or 22q, including neurofibromatosis type 2 (NF2), has been established [19]. Studies have shown that the epithelioid subtype confers a more favorable overall survival (OS) compared to sarcomatoid and biphasic subtypes [20].

### 4.1. Molecular Profiling and Future Personalized Approach to Therapy

The molecular pathogenesis of PPM is poorly understood. Inactivation of BAP1 is frequent in PPM, seen in up to 79% of cases [21]. Some evidence shows that BAP1 mutations are associated with improved survival and may serve as predictive biomarkers for immunotherapy [21,22]. Other genetic alterations identified included cyclin-dependent kinase inhibitor 2A (CDKN2A) deletions (29%), neurofibromin 2 (NF2) (35%), and anaplastic lymphoma kinase (ALK) gene rearrangements (13%) [19,21,22]. Interestingly, ALK-rearranged PPM does not feature BAP1 or NF2 alterations and may be a potential target for ALK-targeted tyrosine kinase inhibitors [19].

Moreover, owing to the insidious nature of the disease, it is difficult to reliably suspect PPM clinically at the initial stages [23]. When the disease progresses to an advanced stage, the most common clinical presentation of PPM includes increased abdominal girth, abdominal pain, nausea, weight loss, and bowel obstruction [7]. Similar to clinical presentation, suspecting PPM on abdominal imaging in isolation is exceptionally challenging. Abdominal compute tomography (CT) findings are nonspecific, ranging from peritoneum-based masses to ascites with associated peritoneal thickening and scalloping of adjacent abdominal organs. Unlike pleural mesotheliomas, calcified peritoneal plaques are rare [24]. Nonetheless, abdominal CT has traditionally been used more frequently than any other imaging modality for disease extent determination in patients with PPM [9].

Ultimately, the gold standard method for PPM diagnosis is tissue biopsy using immunohistochemical staining [25]. The initial panel usually contains two mesothelial markers (cytokeratin 5/6, calretinin, Wilms tumor 1 (WT-1), and D2-40) and two epithelial markers (MOC-31 and claudin-4). After confirmation of the mesothelial lineage, BAP1 loss, CDKN2A homozygous deletion, and MTAP loss were the most specific markers for the diagnosis of malignant mesothelioma. 5-hmC loss and increased EZH2 expression are novel markers for the diagnosis of malignant mesothelioma but are not widely used yet [26,27,28].

The type of therapy employed is determined by the patient clinical status and spread of the disease [29]. Cytoreductive surgery with HIPEC remains the gold standard for suitable candidates, and for those who cannot tolerate surgical resection, systemic chemotherapy alone can be considered [11,29,30]. However, the long-term benefits of systemic chemotherapy alone are not well understood. To the best of our knowledge, no uniformly accepted guidelines are available for radical resection in PPM patients; however, surgical resection is considered in most PPM patients, with no extraperitoneal spread [31]. Likewise, adjuvant radiation therapy is unlikely to provide survival benefits in PPM patients [31,32]. PPM develops in the anatomical region amidst several vital organs, and radiation therapy may add to additional organ damage in these patients [33]. Our study showed that patients receiving surgical intervention had a more favorable five-year survival rate than those receiving either chemotherapy or radiation therapy alone; however, no statistical significance was observed after analyzing the data.

Optimal cytoreductive surgery (CRS) followed by HIPEC is the gold standard for fitting patients without extraperitoenal spread. For patients who are not candidates for CRS/HIPEC, debulking surgery is not routinely performed and systemic chemotherapy is preferred. Pemetrexed in combination with cisplatin, carboplatin, or gemcitabine is preferred for systemic use. No targeted agents or immunotherapies have been specifically approved for PPM treatment. Based on preclinical models, pharmacological inhibition of the PI3K-PTEN-AKT-mTOR pathway has been tested in phase I/II trials [34]. Agents targeting novel molecular pathways and targets, such as mesothelin, vascular endothelial growth factor, histone deacetylase, focal adhesion kinase, and anaplastic lymphoma kinase, are being actively explored [34]. Although immune checkpoint inhibitors are approved for pleural mesothelioma, trials with these agents do not include patients with PPM. Many clinicians use checkpoint inhibitors off-label for MPM, especially PD-1/PDL-1 antibodies, based on responses in single-arm phase I/II trials. A randomized phase IIb trial with tremelimumab, a CTLA4 antibody (DETERMINE), in PPM was negative [35]. While whole abdominal radiation is part of the traditional treatment paradigm for diffuse MPM, radiation has been vanishingly rare in modern times due to its increased toxicity and dubious survival benefit, as evident in our study. Owing to the lack of specific genomic data and treatment details, we were unable to discuss the use of novel or experimental agents in our group of patients.

### 4.2. Limitations

Despite these findings, our study has limitations that are applicable to most database-based studies. Information regarding the timing of chemotherapy relative to surgical resection (adjuvant vs. neoadjuvant), type of chemotherapy (HIPEC vs. systemic chemotherapy), specific agents used for each modality, extent and nature of surgical resection, and radiotherapy dosing schedule were not provided in the SEER database, limiting our interpretation of the results. The outcome data of our study is divided into subgroups of patients who received each modality of treatment, but we do not know whether each treatment modality was used alone or in combination. Some of the critical clinical factors, such as socioeconomic factors, performance status, comorbidities, sub-classification of epithelioid mesothelioma, peritoneal cancer index, mitotic tumor index, and other associated pathologies that might affect the interpretation of the results, are not coded correctly in the SEER database. Finally, the side-effects and complications of surgery, chemotherapy, and radiation therapy were not available in the SEER database. Despite these limitations, our study attempts to adequately describe the clinical and demographic aspects of PPM patients.

Ongoing clinical trials on primary peritoneal mesothelioma (PPM) from National Institute of Health (NIH), United States (Table 7).

## 5. Conclusions

PPM is an aggressive malignancy of the peritoneal surface, where tumor size, Caucasian race, and advanced SEER stage of the disease correlated with poor survival in our study. Surgical resection and HIPEC offers optimum management, with systemic chemotherapy being an option for nonsurgical candidates, and radiation therapy has a limited role in treating patients with PPM. To the best of our knowledge, the current cohort is the most extensive database study of this rare entity. Although the disease is rare; we were able to use a national registry to obtain a substantial number of cases. With the advent of novel agents, further analysis is required to account for changes in the prognosis of various histopathologic subtypes. We strongly suggest that an international registry enrolling all patients with PPM should be introduced to better understand this rare disease.

## Figures and Tables

**Table 1:** Demographic Profiles of 1998 Patients with Primary Peritoneal Mesothelioma from the Surveillance, Epidemiology, and End Results (SEER) database, 1975–2016.

Variable		Frequency (*n*)	Percentage (%)	*p*-Value
Total		1998		
Age (Y)	<50	379	19	<0.005
	>50	1619	81	
Gender	Male	1122	56.2 %	<0.005
	Female	876	43.8 %	
Race	Caucasians	1806	90.4	<0.005
	African Americans	101	5	
	Others	85	4.3	
	Unknown	6	0.3	

Abbreviations: *n* = number; Y = years; % = percentage.

**Table 2:** Tumor Characteristics of 1998 Patients with Primary Peritoneal Mesothelioma from the Surveillance, Epidemiology, and End Results (SEER) database, 1975–2016.

Variable		Frequency (*n*)	Percentage (%)	*p*-Value
Total		1998		
Grade of Differentiation	Well differentiated: Grade 1	106	5.3	<0.005
	Moderately differentiated: Grade 2	37	1.9	
	Poorly differentiated: Grade 3	173	8.7	
	Undifferentiated: Anaplastic: Grade 4	33	1.7	
	Unknown	1649	82.4	
Histological Variant	Mesothelioma, malignant NOS	1266	63.4	<0.005
	Fibrous Mesothelioma, malignant	48	2.4	
	Epithelioid Mesothelioma, malignant	616	30.8	
	Biphasic, Mesothelioma, malignant	67	3.3	
	Multicystic Mesothelioma Malignant	1	0.05	

Abbreviations: *n* = number; % = percentage; NOS = not otherwise specified.

**Table 3:** Tumor stage, size, and lymph node characteristics of 1998 Patients with Primary Peritoneal Mesothelioma from the Surveillance, Epidemiology, and End Results (SEER) database, 1975–2016.

Variable		Frequency (*n*)	Percentage (%)	*p*-Value
Total		1998		
Stage	Localized	166	8.30	<0.001
	Regional	182	9.10	
	Distant	591	29.60	
	Unstaged	170	8.50	
	Unknown	889	44.50	
Tumor Size	Unknown	1785	89.30	<0.005
	<2 cm	21	1.05	
	2–4 cm	32	1.60	
	>4 cm	160	8	
Lymph Nose Status	Nodes Negative	1431	71.60	<0.001
	Nodes positive	122	6.10	
	Unknown	445	22.30	

Abbreviations: *n* = number; cm = centimeters; % = percentage.

**Table 4:** Treatment of 1998 Patients with Primary Peritoneal Mesothelioma from the Surveillance, Epidemiology, and End Results (SEER) database, 1975–2016.

Variable	Frequency (*n*)	Percentage (%)	*p*-Value
Chemotherapy only	1010	56.4	<0.001
Surgery alone	584	29.2	
No treatment data	375	18.7	
Radiation only	29	1.5	

Abbreviations: *n*= number; % = percentage.

**Table 5:** Overall and Treatment Associated Survival Analysis of 1998 Patients with Primary Peritoneal mesothelioma from the Surveillance, Epidemiology, and End Results (SEER) database, 1975–2016.

Variable	Percentage (%)
Overall Survival	
1 year	46.5% ± 1.3
2 year	34.2% ± 1.3
3 year	26.7% ± 1.2
4 year	22.7% ± 1.2
5 year	20.3% ± 1.1
Any Surgery	
1 year	73.5% ± 6.9
2 year	62.2% ± 9.7
3 year	54.5% ± 9.8
4 year	49.7% ± 8.4
5 year	43% ± 5.9
Any Chemotherapy	
1 year	52.8% ± 1.9
2 year	38% ± 1.9
3 year	27.1% ± 1.8
4 year	21.5% ± 1.7
5 year	18.7% ± 1.6
Any Radiation	
1 year	59.3% ± 9.5
2 year	44.4% ± 9.6
3 year	29.6% ± 8.8
4 year	25.9% ± 8.4
5 year	25.9% ± 8.4

Abbreviations: % = percentage.

**Table 6:** Multivariable analysis of factors influencing mortality in patients with Primary Peritoneal mesothelioma from the Surveillance, Epidemiology, and End Results (SEER) database, 1975–2016.

Variables	Odds Ratio (95% C.I.)	*p*-Value
Poorly differentiated	4.2 (3.3–4.9,)	<0.001
Tumor size > 4 cm	3.9 (3.2–4.5)	
Caucasian race	2.9 (2.6–4.4)	
Distant SEER stage	2.5 (1.1–3.2)	

Abbreviations: % = percentage.

**Table 7:** Selected ongoing treatment trials in primary peritoneal mesothelioma. Source: Clinicaltrials.gov, accessed 1 February 2022.

Trial Number	Study Title	Study Type	Intervention	Primary Outcome	Status
NCT03875144 (MESOTIP)	Treatment of malignant peritoneal mesothelioma	Phase 2, randomized, open label	Pressurized intraperitoneal aerosol chemotherapy of cisplatin + doxorubicin vs. systemic cisplatin + pemetrexed	OS	Recruiting
NCT05041062	A study of immunotherapy drugs nivolumab and ipilimumab in patients with resectable malignant peritoneal mesothelioma	Phase 2, open label, single arm	Ipilumumab and nivolumab	Major pathologic response rate	Not recruiting
NCT05001880	Chemotherapy with or without immunotherapy for peritoneal mesothelioma	Phase 2 Randomized	Atezolizumab + bevacizumab + carboplatin + pemetrexed followed by CRS and HIPEC or atezolizumab and bevacizumab vs bevacizumab + carboplatin + pemetrexed followed by CRS and HIPEC or atezolizumab with or without bevacizumab	Response rate	Recruiting
NCT04462809 (TALAMESO)	Efficacy of a maintenance treatment with talazoparib following first line platinum-based chemotherapy in malignant mesothelioma	Phase 2, multiple cohorts	Talazoparib maintenance for two years after surgery and chemotherapy	Non-progression proportion	Not recruiting
NCT04847063	Individualized response assessment to heated intraperitoneal chemotherapy for the treatment of peritoneal carcinomatosis from ovarian, colorectal, appendiceal, or peritoneal mesothelioma histologies	Phase 1, open label	HIPEC with intraperitoneal oxaliplatin and IV 5-FU vs. HIPEC with intraperitoneal mitomycin C vs. HIPEC with intraperitoneal cisplatin and doxorubicin, in addition to IV sodium thiosulfate vs HIPEC with intraperitoneal cisplatin and mitomycin C, in addition to IV sodium thiosulfate	Correlation between ex vivo simulated HIPEC and in vivo HIPEC with respect to two measures of treatment: percent necrosis and Ki-67	Recruiting
NCT00996385	Velcade and eloxatin for patients with malignant pleural or peritoneal mesothelioma	Phase 2, open label	Bortezomib + oxaliplatin	Objective tumor response rate	Unknown
NCT00024271	Surgery, chemotherapy, and radiation therapy in treating patients with peritoneal cancer	Phase 2	Surgery + intraperitoneal chemotherapy with doxorubicin, cisplatin & gemcitabine and intraperitoneal interferron gamma + radiation	N/A	Not recruiting
NCT02399371	Pembrolizumab in treating patients with malignant mesothelioma	Phase 2	Pembrolizumab	Ability of PD-L1 to predict response	Not recruiting
NCT02535312	Methoxyamine, cisplatin, and pemetrexed disodium in treating patients with advanced solid tumors or mesothelioma that cannot be removed by surgery or mesothelioma that is refractory to pemetrexed disodium and cisplatin or carboplatin	Phase 1/2	Methoxyamine + pemetrexed disodium + cisplatin vs. methoxyamine + pemetrexed disodium	Dose-limiting toxicity, response rate	Not recruiting
NCT03054298	CAR T-cells in mesothelin expressing cancers	Phase 1	Lentiviral transduced human CART-cells against mesothelin with or without lymphodepletion	Treatment-related adverse events	Recruiting
NCT03907852	Phase 1/2 trial of gavo-cel (TC-210) in patients with advanced mesothelin-expressing cancer	Phase 1/2	Lymphodepletion followed by gavo-cel (CAR-T cells against mesothelin)	Recommended phase 2 dose and efficacy	Recruiting
NCT04000906 (Nab-PIPAC)	PIPAC with nab-paclitaxel and cisplatin in peritoneal carcinomatosis	Phase 1b	PIPAC administration of nab-paclitaxel and cisplatin	Maximum tolerated dose	Recruiting

Abbreviations; OS, overall survival; CRS, cytoreductive surgery; HIPEC, hyperthermic intraperitoneal chemotherapy; vs., versus; IV, intravenous; 5-FU, 5-fluorouracil; PD-L1, programmed death-ligand 1; N/A, not available; CAR-T, chimeric antigen receptor modified T; gavo-cel, gavocabtagene autoleucel; PIPAC, pressurized intraperitoneal aerosol chemotherapy.

## Data Availability

All data is publicly available. The data of this manuscript was presented at Society of American Gastrointestinal and Endoscopic Surgeons (SAGES), Los Vegas, Nevada, 31 August–3 September 2021.
